# Supplementary material for: Risk factors for graft loss and death among kidney transplant recipients: A competing risk analysis
Source: PLoS One. 2022 Jul 14;17(7):e0269990. doi: 10.1371/journal.pone.0269990 (PMC9282472; doi:10.1371/journal.pone.0269990)
Supplement: S1 File — (DOCX) [file pone.0269990.s001.docx]

**Supplementary material**

**S Table 1. List of predictors**

**Data fields for the study**

| **No** | **Data field** |
| --- | --- |
| **Recipient Characteristics** | |
| 1 | Date of transplantation |
| 2 | Age at transplant |
| 3 | Sex |
| 4 | Social stratification (Relative Socio-economic Disadvantage) |
| 5 | Social support |
| 6 | Body mass index |
| 7 | Zone |
| 8 | Blood group |
| 9 | Primary renal disease |
| 10 | Co-morbid conditions at the entry |
| 11 | - Cardiovascular |
| 12 | - Stroke |
| 13 | - Myocardial revascularization |
| 14 | - Cigarette smoking |
| 15 | - Hypertension |
| 16 | - Diabetes |
| 17 | - Coronary stent |
| 18 | - Chronic Lung (current) |
| 19 | - Coronary Artery (current) |
| 20 | - Peripheral Vascular (current) |
| 21 | - Cerebro Vascular (current) |
| 22 | - Diabetes (current) |
| 23 | - Other comorbid conditions (Current) |
| 24 | Center of treatment : hospital / centre name (Current) |
|  | HLA mismatch |
| 25 | - HLA-A mismatch |
| 26 | - HLA-B mismatch |
| 27 | - HLA-DR mismatch |
| 28 | - HLA-DQ mismatch |
| 29 | - PRA I qualitative |
| 30 | - PRA II qualitative |
| 31 | - PRA I quantitative |
| 32 | - PRA II quantitative |
| 33 | Cancer ever |
|  |  |
|  | **Dialysis data** |
| 34 | Total number months in dialysis |
| 35 | Type of renal replacement therapy (hemodialysis, peritoneal, pre-dialysis) |
| 36 | Time on waiting list |
|  |  |
|  | **Transplant related data** |
| 37 | Graft number |
|  | Recipient antibody status |
| 38 | - CMV |
| 39 | - EBV |
| 40 | - Hep C |
| 41 | - BK virus |
| 42 | Number of rejection episodes |
| 43 | Graft failure date |
| 44 | Graft failure cause |
| 45 | Cause of death |
| 46 | Cold ischemia time |
| 47 | Number of renal allograft biopsies (during follow-up) |
| 48 | Serum creatinine at 12 months |
| 49 | Serum creatinine at 24 months |
| 50 | Serum creatinine at 36 months |
| 51 | Serum creatinine at 48 months |
| 52 | Serum creatinine at 60 months |
| 53 | Re-admissions during follow-up |
| 54 | Acute tubular necrosis |
| 55 | Proteinuria |
|  |  |
| **Donor characteristics** | |
| 56 | Type of donor (deceased, living) |
| 57 | HLA type |
|  | Virology status |
| 58 | - Hep C |
| 59 | - Hep B |
| 60 | - CMV |
| 61 | - EBV |
| 62 | - HIV |

**Supplementary material**

**Modelling process**

#cargar librerias

library(riskRegression)# for Fine-Gray Regression (FGR)
library(prodlim) # for Hist function
library(lava) # not sure, but its needed
library(cmprsk) # competing risks
library(crrstep) # for variable selection in FGR
library(pec) # for resampling metrics

#covariables perdida

fmla_perdida <- as.formula( ~ time5 + status + preACV + TXpraq2 + postx_cantidad_biopsia+
 + postx_nefropatia_BK + postx_rechazo_agudo+ cs12

 )

# perdida remove intercept
bmtcrr.expanded_perdida <- as.data.frame(model.matrix(fmla_perdida,
 data = base_RC_febrero)[,-1])

head(bmtcrr.expanded_perdida)

## time5 status preACV1 TXpraq21 TXpraq23 postx_cantidad_biopsia
## 1 3.38 0 0 1 0 4
## 2 4.48 2 0 0 1 8
## 4 5.00 0 0 0 1 6
## 5 1.55 1 0 0 1 6
## 6 1.75 2 0 1 0 6
## 7 5.00 0 0 0 1 6
## postx_nefropatia_BK1 postx_rechazo_agudo1 cs12
## 1 1 1 2.29
## 2 0 1 1.51
## 4 0 1 0.83
## 5 1 1 2.55
## 6 0 1 1.18
## 7 0 1 1.55

# perdida get the names of the covariates only
covariates_perdida <- setdiff(colnames(bmtcrr.expanded_perdida),
 c("time5","status"))

# perdida formula for the Full model
(ff_perdida <- as.formula(paste0("Hist(time5, status) ~ ",
 paste(covariates_perdida, collapse = "+"))))

## Hist(time5, status) ~ preACV1 + TXpraq21 + TXpraq23 + postx_cantidad_biopsia +
## postx_nefropatia_BK1 + postx_rechazo_agudo1 + cs12

# Fit full model with Fine-Grey regression model
fg_perdida <- riskRegression::FGR(ff_perdida, cause = 2, data = bmtcrr.expanded_perdida)

summary(fg_perdida)

## Competing Risks Regression
##
## Call:
## riskRegression::FGR(formula = ff_perdida, data = bmtcrr.expanded_perdida,
## cause = 2)
##
## coef exp(coef) se(coef) z p-value
## preACV1 2.162 8.685 0.6883 3.14 1.7e-03
## TXpraq21 -1.291 0.275 0.2940 -4.39 1.1e-05
## TXpraq23 -1.961 0.141 0.3096 -6.34 2.4e-10
## postx_cantidad_biopsia 0.247 1.280 0.0833 2.96 3.1e-03
## postx_nefropatia_BK1 1.601 4.960 0.3957 4.05 5.2e-05
## postx_rechazo_agudo1 1.053 2.867 0.3730 2.82 4.7e-03
## cs12 0.576 1.779 0.0631 9.14 0.0e+00
##
## exp(coef) exp(-coef) 2.5% 97.5%
## preACV1 8.685 0.115 2.2535 33.472
## TXpraq21 0.275 3.637 0.1545 0.489
## TXpraq23 0.141 7.109 0.0767 0.258
## postx_cantidad_biopsia 1.280 0.781 1.0870 1.507
## postx_nefropatia_BK1 4.960 0.202 2.2840 10.773
## postx_rechazo_agudo1 2.867 0.349 1.3802 5.955
## cs12 1.779 0.562 1.5725 2.014
##
## Num. cases = 959
## Pseudo Log-likelihood = -325
## Pseudo likelihood ratio test = 171 on 7 df,

# perdida Backward selection based on the AIC
sfgAIC_perdida <- pec::selectFGR(ff_perdida, cause = 2, data = bmtcrr.expanded_perdida,
 rule = "AIC", direction = "backward")
summary(sfgAIC_perdida)

## Length Class Mode
## fit 6 FGR list
## In 3 -none- character
## call 6 -none- call

# perdida Final FGR-model with selected variables
sfgAIC_perdida$fit

##
## Right-censored response of a competing.risks model
##
## No.Observations: 959
##
## Pattern:
##
## Cause event right.censored
## 1 65 0
## 2 64 0
## unknown 0 830
##
##
## Fine-Gray model: analysis of cause 2
##
## Competing Risks Regression
##
## Call:
## riskRegression::FGR(formula = Hist(time5, status) ~ postx_cantidad_biopsia +
## postx_nefropatia_BK1 + cs12, data = data, cause = cause)
##
## coef exp(coef) se(coef) z p-value
## postx_cantidad_biopsia 0.373 1.45 0.0628 5.94 2.8e-09
## postx_nefropatia_BK1 1.489 4.43 0.4008 3.71 2.0e-04
## cs12 0.563 1.76 0.0652 8.64 0.0e+00
##
## exp(coef) exp(-coef) 2.5% 97.5%
## postx_cantidad_biopsia 1.45 0.689 1.28 1.64
## postx_nefropatia_BK1 4.43 0.226 2.02 9.72
## cs12 1.76 0.569 1.55 2.00
##
## Num. cases = 959
## Pseudo Log-likelihood = -347
## Pseudo likelihood ratio test = 128 on 3 df,
##
## Convergence: TRUE

# Backward selection based on the BIC
sfgBIC_perdida <- pec::selectFGR(ff_perdida, cause = 2, data = bmtcrr.expanded_perdida,
 rule = "BIC", direction = "backward")

# Final FGR-model with selected variables
sfgBIC_perdida$fit

##
## Right-censored response of a competing.risks model
##
## No.Observations: 959
##
## Pattern:
##
## Cause event right.censored
## 1 65 0
## 2 64 0
## unknown 0 830
##
##
## Fine-Gray model: analysis of cause 2
##
## Competing Risks Regression
##
## Call:
## riskRegression::FGR(formula = Hist(time5, status) ~ postx_cantidad_biopsia +
## postx_nefropatia_BK1 + cs12, data = data, cause = cause)
##
## coef exp(coef) se(coef) z p-value
## postx_cantidad_biopsia 0.373 1.45 0.0628 5.94 2.8e-09
## postx_nefropatia_BK1 1.489 4.43 0.4008 3.71 2.0e-04
## cs12 0.563 1.76 0.0652 8.64 0.0e+00
##
## exp(coef) exp(-coef) 2.5% 97.5%
## postx_cantidad_biopsia 1.45 0.689 1.28 1.64
## postx_nefropatia_BK1 4.43 0.226 2.02 9.72
## cs12 1.76 0.569 1.55 2.00
##
## Num. cases = 959
## Pseudo Log-likelihood = -347
## Pseudo likelihood ratio test = 128 on 3 df,
##
## Convergence: TRUE

#perdida
# create list of models
models_list_perdida <- list(full.model = fg_perdida, selectedAIC = sfgAIC_perdida, selectedBIC = sfgBIC_perdida)

#perdida
#Bootstrap cross-validation performance
## Lower Brier Score is better
## The reference model is without any covariates
set.seed(7)
p2_perdida <- pec::pec(models_list_perdida,
 formula = ff_perdida,
 data = bmtcrr.expanded_perdida,
 B = 5,
 splitMethod = "Boot632")

## Split sample loop (B=5)

## Warning: executing %dopar% sequentially: no parallel backend registered

## 1

## 2

## 3

## 4

## 5

A1_perdida <- pec::cindex(models_list_perdida,
 formula=ff_perdida,
 data=bmtcrr.expanded_perdida,
 eval.times=10)

## Cindex for competing risks

## Warning in pec::cindex(models_list_perdida, formula = ff_perdida, data =
## bmtcrr.expanded_perdida, : 1 eval.times beyond the maximal evaluation time: 5

p2_perdida

##
## Prediction error curves
##
## Prediction models:
##
## Reference full.model selectedAIC selectedBIC
## Reference full.model selectedAIC selectedBIC
##
## Right-censored response of a competing.risks model
##
## No.Observations: 959
##
## Pattern:
##
## Cause event right.censored
## 1 65 0
## 2 64 0
## unknown 0 830
##
## IPCW: cox model
##
## Method for estimating the prediction error:
##
## Bootstrap cross-validation
##
## Type: resampling
## Bootstrap sample size: 959
## No. bootstrap samples: 5
## Sample size: 959
##
## Cumulative prediction error, aka Integrated Brier score (IBS)
## aka Cumulative rank probability score
##
## Range of integration: 0 and time=5 :
##
##
## Integrated Brier score (crps):
##
## IBS[0;time=5)
## Reference 0.044
## full.model 0.056
## selectedAIC 0.053
## selectedBIC 0.053

plot(p2_perdida)


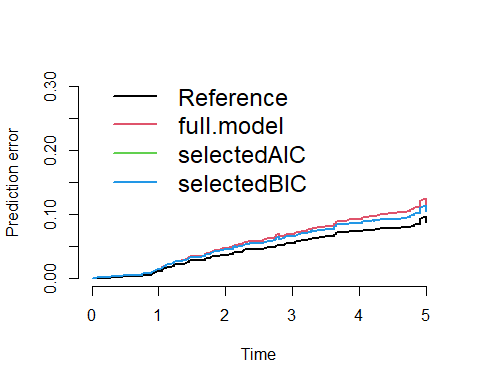
**Fig 3.** **Prediction errors and calibration plot of the final Fine and Gray model for graft loss**

A1_perdida

##
## The c-index for right censored event times
##
## Prediction models:
##
## full.model selectedAIC selectedBIC
## full.model selectedAIC selectedBIC
##
## Right-censored response of a competing.risks model
##
## No.Observations: 959
##
## Pattern:
##
## Cause event right.censored
## 1 65 0
## 2 64 0
## unknown 0 830
##
## Censoring model for IPCW: marginal model (Kaplan-Meier for censoring distribution)
##
## No data splitting: either apparent or independent test sample performance
##
## Estimated C-index in % at time=10
##
## AppCindex Pairs (Di=1,Ti<Tj) Concordant Pairs (Di=1,Dj=2)
## full.model 57.8 41032 25306 2044
## selectedAIC 61.0 41032 27106 2044
## selectedBIC 61.0 41032 27106 2044
## Concordant
## full.model 244
## selectedAIC 291
## selectedBIC 291

## Warning in summary.Cindex(x, print = TRUE, ...): The C-index is not proper for t-year predictions. Blanche et al. (2018), Biostatistics, 20(2): 347--357.
##
## Consider using time-dependent AUC instead: riskRegression::Score

#Calibration Plot
calPlot(models_list_perdida,
 formula=ff_perdida, splitMethod = "BootCv", B=5, bandwidth = 0.01)

**Fig 3. Prediction errors and calibration plot of the final Fine and Gray model for graft loss**


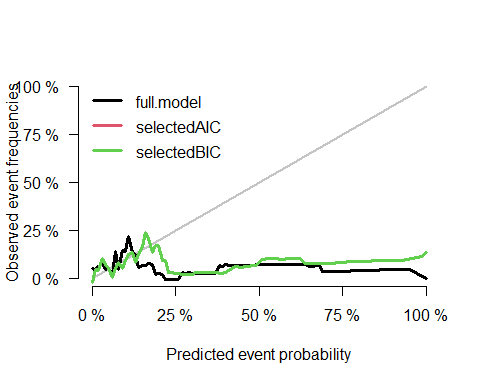


#Observed vs. Predicted for each Method
# Full Model
b1_perdida <- pec::calPlot(models_list_perdida[[1]],
 formula = ff_perdida,
 bars = TRUE,
 hanging = FALSE)
print(b1_perdida)

##
## Calibration of risk predictions for 959 subjects.
##
## Until time 3.01 a total of 479 were observed event-free,
## - a total of 46 were observed to have the event of interest (cause: 1),
## - a total of 42 had a competing risk
## - a total of 392 were lost to follow-up.
##
## Average predictions and outcome in prediction quantiles:
##
## $Model.1
## Pred Obs
## [0.00358,0.0052] 0.004627462 0.04058305
## (0.0052,0.0077] 0.006284435 0.06285348
## (0.0077,0.00945] 0.008690334 0.06795433
## (0.00945,0.011] 0.010151776 0.02222222
## (0.011,0.0137] 0.012110906 0.04476190
## (0.0137,0.0213] 0.017293439 0.07283320
## (0.0213,0.0331] 0.027066998 0.04854366
## (0.0331,0.0519] 0.041750550 0.08437755
## (0.0519,0.107] 0.070312235 0.08212996
## (0.107,1] 0.307259686 0.08037750
##
##
## Outcome frequencies (Obs) were obtained with the Aalen-Johansen method.

plot(b1_perdida)

**S1 Fig. Observed vs. predicted for each method for graft loss.**


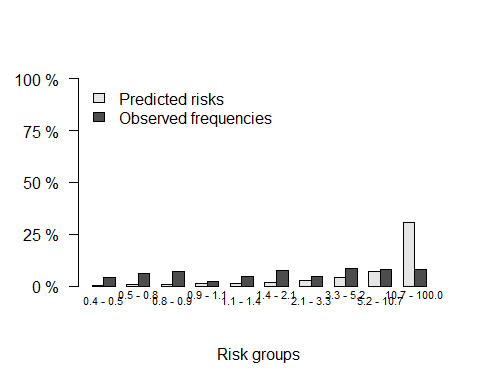


###modelo muerte

# Define training control
set.seed(123)
train.control <- trainControl(method = "cv", number = 10)

#cargar librerias

library(riskRegression)# for Fine-Gray Regression (FGR)
library(prodlim) # for Hist function
library(lava) # not sure, but its needed
library(cmprsk) # competing risks
library(crrstep) # for variable selection in FGR
library(pec) # for resampling metrics

fmla_muerte <- as.formula( ~ time5+status+ edad_trasplante + preimc1 +etiologia+
Terapia_reemplazo_renal+ tiempo_dialisis+
timpodia_recod + tiempoenlista+ preCVDtotal+
preSTENT+ preACV+preREVASCULARIZACION+
preINFARTO+ preHTA+ preDIABETES+ preTABAQUISMO+
pretxcancer+ TX_tipotx+ TXcritexpand+postx_isquemiafria+
tisqrecod+TXinduccion+TX_compA+TX_compB+TX_compDR+missmatch+
match+TXpraq1+TXpraq2+TX_imctx+ postx_estenosis_ureter+
postx_fistulaurinaria+ postx_hematoma_postx+ postx_ISO+
postx_CMV+ postx_nefropatia_BK+ postx_CVD+ poststent+
postx_acv+ postx_revascularizacion+ postx_iam+
postx_diabetes_postx+postx_tabaquismo+postx_Cancer+
postx_biopsia+ postx_cantidad_biopsia+
postx_rechazo_agudo+postx_rechazo_cronico+ postx_NTA+
cs12+ postx_rehospitalizacion+ postx_cantidad_hospitalizaciones)

#covariables muerte
fmla_muerte <- as.formula( ~ time5+status + edad_trasplante + preDIABETES + TX_tipotx+
tisqrecod+
postx_CMV+ poststent+
postx_rechazo_agudo+
postx_cantidad_hospitalizaciones
)

# remove intercept
bmtcrr.expanded_muerte <- as.data.frame(model.matrix(fmla_muerte,
 data = base_RC_febrero)[,-1])

head(bmtcrr.expanded_muerte)

## time5 status edad_trasplante preDIABETES1 TX_tipotxLive tisqrecod1 postx_CMV1
## 1 3.38 0 31 0 1 0 0
## 2 4.48 2 20 0 0 0 1
## 3 5.00 0 27 0 1 0 0
## 4 5.00 0 18 0 0 1 0
## 5 1.55 1 59 0 0 1 1
## 6 1.75 2 21 0 1 0 0
## poststent1 postx_rechazo_agudo1 postx_cantidad_hospitalizaciones
## 1 0 1 4
## 2 0 1 5
## 3 0 1 4
## 4 0 1 7
## 5 0 1 3
## 6 0 1 1

# get the names of the covariates only
covariates_muerte <- setdiff(colnames(bmtcrr.expanded_muerte),
 c("time5","status"))

# formula for the Full model
(ff_muerte <- as.formula(paste0("Hist(time5, status) ~ ",
 paste(covariates_muerte, collapse = "+"))))

## Hist(time5, status) ~ edad_trasplante + preDIABETES1 + TX_tipotxLive +
## tisqrecod1 + postx_CMV1 + poststent1 + postx_rechazo_agudo1 +
## postx_cantidad_hospitalizaciones

# Fit full model with Fine-Grey regression model
fg_muerte <- riskRegression::FGR(ff_muerte, cause = 1, data = bmtcrr.expanded_muerte)

summary(fg_muerte)

## Competing Risks Regression
##
## Call:
## riskRegression::FGR(formula = ff_muerte, data = bmtcrr.expanded_muerte,
## cause = 1)
##
## coef exp(coef) se(coef) z p-value
## edad_trasplante 0.0390 1.040 0.00759 5.142 2.7e-07
## preDIABETES1 0.2716 1.312 0.21494 1.264 2.1e-01
## TX_tipotxLive -0.8019 0.448 0.30913 -2.594 9.5e-03
## tisqrecod1 0.1770 1.194 0.19817 0.893 3.7e-01
## postx_CMV1 0.8619 2.368 0.28121 3.065 2.2e-03
## poststent1 1.0703 2.916 0.55901 1.915 5.6e-02
## postx_rechazo_agudo1 0.2879 1.334 0.18418 1.563 1.2e-01
## postx_cantidad_hospitalizaciones -0.0254 0.975 0.04144 -0.614 5.4e-01
##
## exp(coef) exp(-coef) 2.5% 97.5%
## edad_trasplante 1.040 0.962 1.024 1.055
## preDIABETES1 1.312 0.762 0.861 1.999
## TX_tipotxLive 0.448 2.230 0.245 0.822
## tisqrecod1 1.194 0.838 0.809 1.760
## postx_CMV1 2.368 0.422 1.364 4.108
## poststent1 2.916 0.343 0.975 8.723
## postx_rechazo_agudo1 1.334 0.750 0.930 1.913
## postx_cantidad_hospitalizaciones 0.975 1.026 0.899 1.057
##
## Num. cases = 1454
## Pseudo Log-likelihood = -903
## Pseudo likelihood ratio test = 79.8 on 8 df,

# Backward selection based on the AIC
sfgAIC_muerte <- pec::selectFGR(ff_muerte, cause = 1, data = bmtcrr.expanded_muerte,
 rule = "AIC", direction = "backward")
summary(sfgAIC_muerte)

## Length Class Mode
## fit 6 FGR list
## In 5 -none- character
## call 6 -none- call

# Final FGR-model with selected variables
sfgAIC_muerte$fit

##
## Right-censored response of a competing.risks model
##
## No.Observations: 1454
##
## Pattern:
##
## Cause event right.censored
## 1 137 0
## 2 169 0
## unknown 0 1148
##
##
## Fine-Gray model: analysis of cause 1
##
## Competing Risks Regression
##
## Call:
## riskRegression::FGR(formula = Hist(time5, status) ~ edad_trasplante +
## TX_tipotxLive + postx_CMV1 + poststent1 + postx_rechazo_agudo1,
## data = data, cause = cause)
##
## coef exp(coef) se(coef) z p-value
## edad_trasplante 0.0417 1.043 0.00698 5.98 2.3e-09
## TX_tipotxLive -0.9075 0.404 0.27966 -3.24 1.2e-03
## postx_CMV1 0.8078 2.243 0.26085 3.10 2.0e-03
## poststent1 1.0619 2.892 0.55877 1.90 5.7e-02
## postx_rechazo_agudo1 0.2711 1.311 0.17561 1.54 1.2e-01
##
## exp(coef) exp(-coef) 2.5% 97.5%
## edad_trasplante 1.043 0.959 1.028 1.057
## TX_tipotxLive 0.404 2.478 0.233 0.698
## postx_CMV1 2.243 0.446 1.345 3.740
## poststent1 2.892 0.346 0.967 8.646
## postx_rechazo_agudo1 1.311 0.763 0.930 1.850
##
## Num. cases = 1454
## Pseudo Log-likelihood = -905
## Pseudo likelihood ratio test = 77.1 on 5 df,
##
## Convergence: TRUE

# Backward selection based on the BIC
sfgBIC_muerte <- pec::selectFGR(ff_muerte, cause = 1, data = bmtcrr.expanded_muerte,
 rule = "BIC", direction = "backward")

# Final FGR-model with selected variables
sfgBIC_muerte$fit

##
## Right-censored response of a competing.risks model
##
## No.Observations: 1454
##
## Pattern:
##
## Cause event right.censored
## 1 137 0
## 2 169 0
## unknown 0 1148
##
##
## Fine-Gray model: analysis of cause 1
##
## Competing Risks Regression
##
## Call:
## riskRegression::FGR(formula = Hist(time5, status) ~ edad_trasplante +
## TX_tipotxLive + postx_CMV1 + poststent1 + postx_rechazo_agudo1,
## data = data, cause = cause)
##
## coef exp(coef) se(coef) z p-value
## edad_trasplante 0.0417 1.043 0.00698 5.98 2.3e-09
## TX_tipotxLive -0.9075 0.404 0.27966 -3.24 1.2e-03
## postx_CMV1 0.8078 2.243 0.26085 3.10 2.0e-03
## poststent1 1.0619 2.892 0.55877 1.90 5.7e-02
## postx_rechazo_agudo1 0.2711 1.311 0.17561 1.54 1.2e-01
##
## exp(coef) exp(-coef) 2.5% 97.5%
## edad_trasplante 1.043 0.959 1.028 1.057
## TX_tipotxLive 0.404 2.478 0.233 0.698
## postx_CMV1 2.243 0.446 1.345 3.740
## poststent1 2.892 0.346 0.967 8.646
## postx_rechazo_agudo1 1.311 0.763 0.930 1.850
##
## Num. cases = 1454
## Pseudo Log-likelihood = -905
## Pseudo likelihood ratio test = 77.1 on 5 df,
##
## Convergence: TRUE

# create list of models
models_list_muerte <- list(full.model = fg_muerte, selectedAIC = sfgAIC_muerte, selectedBIC = sfgBIC_muerte)

#Bootstrap cross-validation performance
## Lower Brier Score is better
## The reference model is without any covariates
set.seed(7)
p2_muerte <- pec::pec(models_list_muerte,
 formula = ff_muerte,
 data = bmtcrr.expanded_muerte,
 B = 5,
 splitMethod = "Boot632")

## Split sample loop (B=5)

## 1

## 2

## 3

## 4

## 5

A1_muerte <- pec::cindex(models_list_muerte,
 formula=ff_muerte,
 data=bmtcrr.expanded_muerte,
 eval.times=10)

## Cindex for competing risks

## Warning in pec::cindex(models_list_muerte, formula = ff_muerte, data =
## bmtcrr.expanded_muerte, : 1 eval.times beyond the maximal evaluation time: 5

p2_muerte

##
## Prediction error curves
##
## Prediction models:
##
## Reference full.model selectedAIC selectedBIC
## Reference full.model selectedAIC selectedBIC
##
## Right-censored response of a competing.risks model
##
## No.Observations: 1454
##
## Pattern:
##
## Cause event right.censored
## 1 137 0
## 2 169 0
## unknown 0 1148
##
## IPCW: cox model
##
## Method for estimating the prediction error:
##
## Bootstrap cross-validation
##
## Type: resampling
## Bootstrap sample size: 1454
## No. bootstrap samples: 5
## Sample size: 1454
##
## Cumulative prediction error, aka Integrated Brier score (IBS)
## aka Cumulative rank probability score
##
## Range of integration: 0 and time=5 :
##
##
## Integrated Brier score (crps):
##
## IBS[0;time=5)
## Reference 0.075
## full.model 0.072
## selectedAIC 0.072
## selectedBIC 0.072

plot(p2_muerte)

**Fig 4. Prediction errors and calibration plot of the final Fine and Gray model for death**


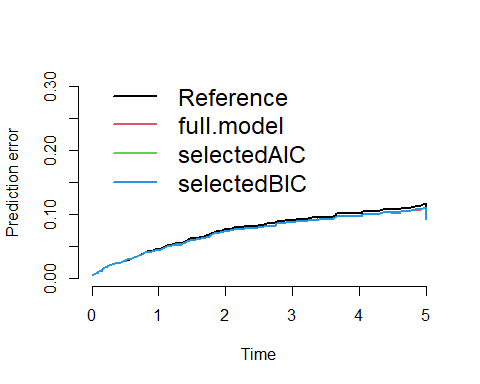


A1_muerte

##
## The c-index for right censored event times
##
## Prediction models:
##
## full.model selectedAIC selectedBIC
## full.model selectedAIC selectedBIC
##
## Right-censored response of a competing.risks model
##
## No.Observations: 1454
##
## Pattern:
##
## Cause event right.censored
## 1 137 0
## 2 169 0
## unknown 0 1148
##
## Censoring model for IPCW: marginal model (Kaplan-Meier for censoring distribution)
##
## No data splitting: either apparent or independent test sample performance
##
## Estimated C-index in % at time=10
##
## AppCindex Pairs (Di=1,Ti<Tj) Concordant Pairs (Di=1,Dj=2)
## full.model 70.8 130266 92840 14047
## selectedAIC 70.4 130266 92100 14047
## selectedBIC 70.4 130266 92100 14047
## Concordant
## full.model 8976
## selectedAIC 8979
## selectedBIC 8979

## Warning in summary.Cindex(x, print = TRUE, ...): The C-index is not proper for t-year predictions. Blanche et al. (2018), Biostatistics, 20(2): 347--357.
##
## Consider using time-dependent AUC instead: riskRegression::Score

#Calibration Plot
calPlot(models_list_muerte,
 formula=ff_muerte, splitMethod = "BootCv", B=5, bandwidth = 0.01)

**S2 Fig. Methods of variable selection.**


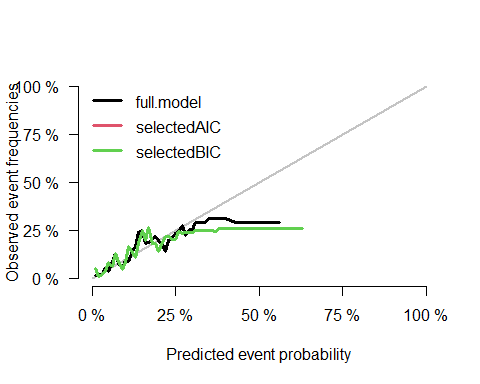


AIC: Akaike selection criteria; BIC: Bayasiani selection criteria.

#Observed vs. Predicted for each Method
# Full Model
b1_muerte <- pec::calPlot(models_list_muerte[[1]],
 formula = ff_muerte,
 bars = TRUE,
 hanging = FALSE)
print(b1_muerte)

##
## Calibration of risk predictions for 1454 subjects.
##
## Until time 2.12 a total of 723 were observed event-free,
## - a total of 102 were observed to have the event of interest (cause: 1),
## - a total of 130 had a competing risk
## - a total of 499 were lost to follow-up.
##
## Average predictions and outcome in prediction quantiles:
##
## $Model.1
## Pred Obs
## [0.00994,0.0197] 0.01540092 0.01001818
## (0.0197,0.0305] 0.02513139 0.01488296
## (0.0305,0.0415] 0.03657068 0.02763084
## (0.0415,0.0537] 0.04752704 0.03984398
## (0.0537,0.0672] 0.06080850 0.07191762
## (0.0672,0.084] 0.07532326 0.12187947
## (0.084,0.102] 0.09220362 0.08410092
## (0.102,0.126] 0.11557661 0.07988406
## (0.126,0.168] 0.14566397 0.17418056
## (0.168,0.651] 0.23018881 0.22290343
##
##
## Outcome frequencies (Obs) were obtained with the Aalen-Johansen method.

plot(b1_muerte)

**S3 Fig. Observed vs. predicted for each method for**
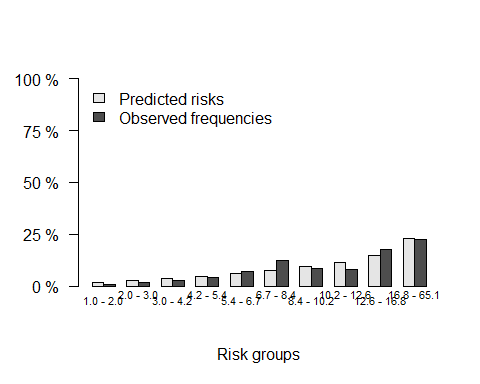
**death**
