# Supplementary material for: Risk factors for graft loss and death among kidney transplant recipients: A competing risk analysis
Source: PLoS One. 2022 Jul 14;17(7):e0269990. doi: 10.1371/journal.pone.0269990 (PMC9282472; doi:10.1371/journal.pone.0269990)
Supplement: Attachment — Submitted filename: Response Letter.docx [file pone.0269990.s002.docx]

Bogotá DC, Abril 1th 2022

Dear Doctor,

**Emily Chenette, PhD**

**Editor-in-Chief**

**PLOS ONE**

**Response Letter**

We summarized the corrections of the research manuscript “Risk factors for graft loss and death among kidney transplant recipients: A competing risk analysis”, please see below:

Journal requirements

1. We ensured our manuscript met PLOS ONE's style requirements.
2. Ethics statement: None of the transplant donors was from a vulnerable population and all donors or next of kin provided written informed consent that was freely given.
3. We provided the original and the English version of the “Organ and Tissue Donation Consent Form” from deceased donors.
4. The participant recruitment dates and the period during which transplant procedures were done are provided in the methods section.
5. In the methods section is specified thar “the total of donor medical costs is covered including organ donor maintenance and procurement with an average of 5000 USD and without any economic contribution to the family donor”.
6. We provided a repository of information for our data that is available with DOI: 10.17632/3j2m4ftn69.1

Reviewer 1

Abstract:

- We changed the conclusion

Introduction:

1. CKD epidemiology was removed from the manuscript.
2. We corrected the paragraph in lines 78-79 about death as a competing event to graft loss.
3. We did not use the Competing Random Forest (CRF) to analyze our data. Instead, we used the Cumulative Incidence Function (CIF).

Methods

4. Line 152: We spelled out The Akaike information criterion (AIC) and the Bayesian Information Criterion (BIC).
5. Tables 1 and 2: We added the p values that are significant in Table 1, and we removed further Tables that were all summarized in one table.
6. Table 2: Table 2 was removed
7. Table 2: We did not have any TG or IFTA data available to analyze the effect on graft survival. Table 2 was removed.
8. Tables 3 and 4: Our center does not perform protocol biopsies. We perform kidney biopsies only with clinical indications. Thus, this could be the reason for the correlation between the number of biopsies and graft loss (indication bias). Tables 3 and 4 were summarized in Table 2.
9. We do not have KDPI available for donors in our country.
10. 1)We included only pretransplant DM, 2) We did not have available data about the proportion of DM type 1 and 2.

Discussion:

11. Line 220: We added reference 9 and consolidate it.
12. Line 243: We included the articles suggested about BK nephropathy and graft loss.
13. Paragraph 260-266: in the methods section we specified how were collected the potential risk factors for graft loss, we included those published from the existing literature and also those available in our data. Those predictors not included are recognized as a limitation.
14. Why DM did not remain an independent risk factor of death (contrary to other studies)? We think that in the case of diabetes the sample size was not sufficient

Reviewer 2

1. Since the KM model does not account for competing risk, we removed the KM model.
2. We changed the conclusion and focused it on the aim of this paper which is to identify risk factors.
3. Propensity scores are used to balance observed covariates between subjects from the study groups to mimic the situation of a randomized trial (Joffe & Rosenbaum, 1999) and can be used for matching, stratification, or in a regression model as a covariate or weight (Rubin, 1997; D’Agostino, 1998). Because propensity scores are used to address potential confounding by indication, they would not be expected to improve pure prediction, which is not concerned with specific coefficient estimation. Additionally, propensity scores are estimated from regressions that comprise the same covariates included in the traditional prediction models, and only those covariates, thus it would seem mathematically impossible for the propensity scores to add anything – they are simply functions of the same variables already included in the traditional models. Despite this argument, requests for the addition of propensity scores to pure prediction models persist <https://www.ncbi.nlm.nih.gov/pmc/articles/PMC3740143/>
4. We specified how the final models were selected in the methods section.
5. We used “univariable/multivariable” instead of “univariate/multivariate”.
6. We cited the reference to the competing risk analysis.
7. We Added p values to Table 1 and 2. (now summarized in table 1)
8. Table 3 footnote: “IC” should be “CI”. (now in table 2)
9. We removed The “Model performance” section from the results

Reviewer 3

Major 
 1. We emphasized the development of prediction models in the INTRODUCTION and DISCUSSION sections of the article.

2. We consulted and used STROBE and TRIPOD statements

ABSTRACT: 
3. We clarified the predictor variable definition and handling, as well as the variable selection process for each model depending on outcome in the methods section 
4. The main conclusion was improved to be aligned with the main objective(s) of the manuscript. 
 
INTRODUCTION: 
5. We reviewed previously published prediction models for similar outcomes in kidney transplant recipients and the important predictors identified from those. 
 
METHODS: 
6. We mentioned protocols for treating rejection 
7. We discussed HLA (in)compatibility assessment pre-transplant and how it affects allocation decisions.

8. We provided the list of predictors and variables in a supplementary material. We also clarified predictors in the method section
9. Multiple imputation for handling missing values was no performed as there were few missing values
10. We specified the Modelling technique and predictor selection

11. We considered LASSO for fitting parsimonious prediction models. Unfortunately, for the package crrp:::crrp  (this package is useful for penalized Fine-Gray models, using LASSO, SCAD, MCP, and their group versions) there is no predict function, and the output from the crrp() function does not include convenient parameters such as a P value. There are standard errors, so a P value can be manually calculated. Creating a tidy wrapper may be difficult. In addition, this package has not been maintained since its first commit in 2015.

12. Analysis of time-dependent covariates has some limitations in both the survival analysis and the competing risks setting. When different causes are acting simultaneously, the main interest is in estimating quantities such as cause-specific hazards, cumulative incidences or marginal survival probabilities. If random (internal) time-dependent covariates are to be included in the modeling process, then it is still possible to estimate cause-specific hazards but prediction of the cumulative incidences and survival probabilities based on these is no longer feasible (Andersenet al., 1993, Chapter VII; Kalbfleisch and Prentice, 2002, Chapter 6). This limitation is also encountered in the regression approach for cumulative incidences (or ‘‘subdistribution hazards’’) (Fine and Gray, 1999) and has been further discussed by Latouche, Porcher, and Chevret (2005) and Latoucheet al.(2007) and tackled by Beyersmann and Schumacher (2008)
 
13. We specified the timeframe the model’s intent to prognosticate for in the methods section (5 years postrasplant)
 14. Internal validation of the models was performed.

RESULTS:

15. Table 3 was removed.
16. Biological sex was considered among the predictor variables, not gender.
17. Figure 3, is now figure 2. We change the scale of figures.
18. We indicated that figure 3 (now figure 2) shows CIF of graft failure and death
19. The model performance section is explained in the methods section.
20. The model discrimination and calibration were reported
21. Characteristics of modelling are provided in a supplementary material

 
DISCUSSION: 
- We summarized the main novel observations in the first paragraph

-We discussed the limitations of stepwise regression selection.

- We performed internal validation using resampling techniques.

Minor

1. Line 95: We replaced “competitive” with “competing”.
2. Line 134: We replaced “available case analysis” with “complete case analysis”.
3. We defined PRA II negative in Table 1.
4. We corrected the observation about BMI in Table 2 which is related to the post-transplant recipient BMI (Now summarized in Table 1).
5. We gave a definition of a match.
6. We added the Banff classification for rejection diagnoses.
